# Sex proportion as a covariate increases the statistical test power in growth performance based experiments using as-hatched broilers

**DOI:** 10.1371/journal.pone.0280040

**Published:** 2023-01-20

**Authors:** Ashley D. England, Sosthene Musigwa, Alip Kumar, Ali Daneshmand, Kosar Gharib-Naseri, Sarbast K. Kheravii, Gene Pesti, Shu-Biao Wu

**Affiliations:** 1 School of Environmental and Rural Science, University of New England, Armidale, New South Wales, Australia; 2 Department of Poultry Science, The University of Georgia, Athens, Georgia, United States of America; Universidade Federal de Mato Grosso do Sul, BRAZIL

## Abstract

The availability of sexed day-old broiler chicks is becoming an issue as feather sexing is no longer possible. This has great implications for broiler researchers as the use of randomly distributed mixed-sex birds may result in a greater between-pen variation and thus less statistical power than the use of single-sex birds. The objective of this study was to evaluate the effect of including sex proportion as a covariate in an analysis of covariance (ANCOVA) on the statistical power compared to analysis of variance (ANOVA) where sex was not considered. The statistical parameters examined include mean square error (MSE), the F-statistic, model fit, model significance and observed power. A total of 4 separate experiments that used mixed-sex broilers with unequal numbers of male and female birds per pen were conducted during which performance of the birds was measured. The male % in each pen was recorded during each experiment and corrected for mortality. The performance results were analysed by ANOVA and the statistical parameters were then compared to ANCOVA where sex proportion was included as a covariate. The results showed that a set of assumptions first needed to be met to run ANCOVA. In addition, if the ANOVA results show a high level of model significance and power, then ANCOVA may not be necessary. In other circumstances where the assumptions are met and model significance and observed power are low, the inclusion of sex proportion as a covariate in the analysis will help to reduce MSE, increase the F-statistic value and improve the model significance, model fit and observed power. Therefore, it is suggested that sex proportion should be considered as a covariate in ANCOVA to improve statistical power in nutritional experiments when male and female broilers are unequally and randomly distributed in pens.

## Introduction

The availability of sexed day-old broiler chicks for use in nutritional studies is becoming an increasing issue as feather sexing is no longer possible due to the change in the genetics of the current commercial broiler breeds. This has great implications for broiler researchers as the use of randomly distributed mixed-sex birds may result in greater between-pen variations for the measurements using pen/cage as the replication unit. It is well known that male and female broilers differ in their growth performance with males having a higher body weight gain (BWG), feed intake (FI) and lower feed conversion ratio (FCR) compared to female broilers [1, 2]. This suggests that when researchers use mixed-sex birds to evaluate performance responses to different treatments, variation due to the sex effect may be present in the experimental model thus the model becomes less powerful if sex is not factored in the analysis. In broiler studies, especially for nutritional experiments, analysis of variance (ANOVA) has been used extensively most often with male-only birds and occasionally with mixed-sex birds. However, the inclusion of a covariate, i.e., analysis of covariance (ANCOVA) has been rare even when mixed-sex birds are used without knowing their sex prior to the allocation to the pens/cages [3, 4].

As a statistical analysis method, ANCOVA should be performed only when the data meets a set of assumptions. These assumptions include: the dependent variables should be normally distributed within each treatment group, the variance of the dependent variables must be equal over all treatment groups, there must be homogeneity of regression slopes, and the relation between the covariate and dependent variable must be linear [5]. When experiments using mixed-sex broilers result in a statistically non-significant difference in performance between treatment means by using ANOVA, it may be that the reason for this is variability between the different replicates that is not accounted for in the statistical analysis. Variation due to random effects of choosing chicks at one-day of age and for sampling, and mortality are rarely considered in the analysis model. This variation, or errors, in the ANOVA should not be disregarded or viewed as unexplainable random variation apart from the treatment effects. Different sex proportions should be taken into consideration, for example, to use percentage of males as a covariate, i.e., the use of the ANCOVA model. In this case, the analysis will be able to take into account the error due to the sex effect, thus increasing the power of analysis compared to ANOVA [6].

Another advantage of ANCOVA compared to ANOVA is the bias reduction [7]. For example, in broiler performance studies, male broilers grow faster than females. We would expect mean BWG to be higher in pens that contain more male birds compared to pens containing more female birds. Therefore, there will be a bias created in the groups that happen to have more male birds as they would have a higher BWG than if there was an equal number of males and females in a group. ANCOVA accounts for suspected types of bias by including additional variable(s) in the analysis model. This results in a smaller mean square error (MSE) (see S1–S8 Tables). The MSE is the denominator in the formula to calculate F-values, so a smaller MSE value will result in higher F-values and lower significance probabilities for most variables. The square root of the MSE estimates the standard deviation (SD) of the treatment groups. Dividing the SD by the square root of the total number of observations, minus 1, gives the standard error (SE) of the grand mean. Comparing the SEMs from ANOVA and ANCOVA gives a measure of the effect of the covariate, and dividing the SD by the square root of the number of observations in each treatment group, minus 1, gives the pooled SE.

It is surprising that despite the advantages of ANCOVA, the majority of studies performed using mixed-sex birds did not use sex proportion as a covariate in the statistical analyses. Researchers may not realize the magnitude of sex proportion variation in their studies or not realize that small variations in sex proportions may lower error mean squares enough to lower significance probabilities between their treatments. The proportion of pens in a study with the expected 50:50 sex ratio from randomly chosen chicks is relatively easy to calculate. The odds of getting 5 males and 5 females in a pen are 25%; 10 and 10, 18%; 20 and 20, 13%; 25 and 25, 11%, and so on. Therefore, with a trial with 48 pens of 50 birds each, only about 6 pens would be expected to have the desired ratio if left to chance. All the others would have some bias before random mortalities and random sampling effect the ratios.

The hypothesis of this research is that the differences in sex ratio bias is large enough to have an effect on the outcome of practical experiments with growing broiler chickens at later stages. This was done using the data from 4 experiments. Experiment 1 was performed and reported by Kumar et al. [8] aiming to evaluate the potential of two different types of feed additives to improve performance and intestinal health in broilers as antibiotic alternatives using the clinical necrotic enteritis challenge model. Experiment 2 was part of the study performed and reported by Gharib-Naseri et al. [9]. The aim of this experiment was to evaluate the effect of two different doses of a partially buffered formic acid product and a monoglyceride blend of short and medium-chain fatty acids on necrotic enteritis infected broilers to examine performance, intestinal microbial population and short-chain fatty acids concentrations in the gastrointestinal tract. Experiment 3 was part of the experiment performed and reported by Daneshmand et al. [10] aiming to determine the ability of two feed additives to improve performance and intestinal health in broilers challenged with necrotic enteritis. Experiment 4 was part of the experiment performed and reported by Musigwa et al. [11] and its aim was to investigate the effect of supplementing multicarbohydrases (MC) to broiler diets containing either low (LS), intermediate (IS) or high (HS) soluble arabinoxylan (sAX) to total arabinoxylan (tAX) ratio (sAX/tAX) on energy partitioning, nitrogen (N) balance, and performance.

In experiments 1 and 2, the birds were sexed on d 0 meaning the beginning sex ratios were fixed (subject to feather sexing errors). In the other two experiments, birds were randomly assigned to pens on d 0. It is important to demonstrate the usefulness of ANCOVA under different experimental settings to reduce random error resulting in improved observed power and reduced bias. The current study investigated whether the use of sex proportion as a covariate in ANCOVA is beneficial in improving the statistical power in four experiments, where mixed-sex birds and different treatments were applied, and the circumstances when ANCOVA may not be necessary.

## Materials and methods

### Animal ethics

All experiments were approved by the Animal Ethics Committee of the University of New England with approval No. AEC18-007 (experiment 1), AEC17-066 (Experiment 2), AEC19-034 (experiment 3), AEC17-027 (experiment 4). All broiler management procedures including health care, husbandry and use of laboratory animals fulfilled the requirements of the Australian Code for the Care and Use of Animals for Scientific Purposes (NHMRC, 2013).

### Experimental design

#### Experiment 1

A total of 544 mixed-sex Ross 308 chicks were feather sexed upon arrival and allocated according to sex to four treatments in 32-floor pens, based on a completely randomised design (CRD). Each of the four treatment groups had eight replicate pens with 17 birds per pen (8 males and 9 females); this meant that the male % was similar between most of the pens during the starter period (d 0–10). Pen weight and FI were recorded on days 0, 10, 24, and 35. Body weights of dead birds were recorded daily and FCR was corrected for the mortalities. Dead birds, sampled birds, and birds remaining at the end of the study (day 35) were opened to further confirm their sex by visual inspection of the presence of testes. The male % during each phase varied due to mortalities, culls and sampling that took place. On the final day of the experiment (d 35) all birds were opened to record their sex by the visual inspection of the presence of testes and the male % between different pens ranged from 0% to 69.79%. The male % was then calculated for each phase according to the sex of culled and dead birds.

#### Experiment 2

A total of 528 day-old Ross 308 mixed-sex chicks were feather sexed upon arrival and allocated according to sex to 48 pens with 11 birds in each pen (5 males and 6 females); this meant that the male % was similar between most of the pens during the starter period (d 0–10). Six dietary treatments were applied in this study. Pen body weight and FI were recorded on days 0, 10, 24 and 35 and used to calculate mean bird weight gain, FI, and FCR. FCR was corrected for mortality by adding the weight of dead chickens back to the pen body weight within each period. The male % during each phase varied due to the mortalities, culls and sampling that took place. Culled and dead birds were opened for autopsy and sex was recorded. On the final day of the experiment (d 35) all birds were opened to confirm their sex visual inspection of the presence of testes. The male % between different pens ranged from 11.11% to 62.50%. The male % was then calculated for each phase according to the sex of culled and dead birds.

#### Experiment 3

A total of 816 day-old mixed-sex Cobb 500 chicks were randomly allocated to 48 pens with 17 birds per pen. Six dietary treatments (positive control, no challenge (T1), negative control, challenge (T2), Amasil NA, challenge (T3), Balangut LS P, challenge (T4), Amasil NA + Balangut LS P, challenge (T5) and Zn bacitracin, challenge (T6) with 8 replicates each were used in a randomised design. Pen weight and cumulative pen FI were recorded on days 0, 9, 16, 21, 28 and 35 and used to calculate mean bird weight, FI, and FCR (corrected for mortality). Culled and dead birds were opened for autopsy and sex was recorded. On the final day of the experiment (d 35) all birds were opened to record their sex by the visual inspection of the presence of testes and the male % between different pens ranged from 16.67% to 91.67%. The male % was then calculated for each phase according to the sex of culled and dead birds.

#### Experiment 4

A 2 × 3 factorial arrangement of treatments (MC, no or yes; and three sAX/tAX ratios; high (HS), intermediate (IS) or low (LS)) was applied to 768 Cobb 500 mixed-sex broilers. The birds were randomly allocated to 48 floor pens containing 16 birds/pen. Records for feed and bird weight were obtained on d 19, 28 and 35. The male % during each phase varied due to the randomization, mortalities, culls and sampling that took place. Culled and dead birds were opened for autopsy and sex was recorded. On the final day of the experiment (d 35) all birds were opened to record their sex by the visual inspection of the presence of testes and the male % between different pens ranged from 23.7% to 83.7%. The male % was then calculated for each phase according to the sex of culled and dead birds.

### Calculation of the covariate

The percentages of males were calculated according to the periods for the measurements of BWG, FI and FCR. When any mortalities, sampling or culling of the birds took place during the measurement period, the proportion of time that these birds were alive was considered as a proportion of the whole period according to their contributions to the BWG of the whole period by referencing the breed performance objectives [12, 13]. The calculation was done according to equation.

$$\text{N}=\left({\text{BW}}_{\text{d}}–{\text{BW}}_{\text{i}}\right)/\left({\text{BW}}_{\text{f}}–{\text{BW}}_{\text{i}}\right)$$

Where N is the value representing the proportional number of the bird when it was culled, died or sampled; BW_d_, BW_i_, and BW_f_ denote the body weights in the breed performance objectives on the day of culling, mortality, or sampling, the initial day of the measurement period, and the final day of the measurement period. For example, a culled, dead, or sampled Ross 308 male bird on d 15, was counted as: (576–321)/(1225–321) = 0.282, for the period of d 10–24, while it was counted as: (576–43)/(2235–43) = 0.243 during d 0–35. Here, the BW of the birds in the ROSS 308 breed performance objectives are 43, 321, 576 and 2235 g on d 0, 15, 24, and 35, respectively. The male % in each pen was then determined and used as the covariate in the statistical analysis.

### Statistical procedures

The performance data from all four experiments were first tested for outliers and normality distribution. Any outliers were excluded from the final data sets using the outliers function in SPSS with the Tukey Method. The data from experiments 1–3 were then analysed using a one-way ANOVA, and a one-way ANCOVA with male % for each pen included as a covariate, as a completely randomised design using the General Linear Model procedure of SPSS statistics version 22 (IBM Corporation, Armonk, NY, United States). The correlation between the dependent variables and male % was tested by using the bivariate correlation function in SPSS where the Pearson correlation coefficient with two-tailed test of significance was applied.

For the analysis for one-way ANOVA with d 24–35 BWG as an example where BWG (AVWGAIN2435) was defined as the dependent variable and treatments (TRT) as the independent variable, the SPSS Syntax codes are:

UNIANOVA AVWGAIND2435 BY TRT

 /METHOD = SSTYPE(3)

 /INTERCEPT = INCLUDE

 /POSTHOC = TRT(TUKEY T2)

 /EMMEANS = TABLES(OVERALL)

 /EMMEANS = TABLES(TRT) COMPARE ADJ(BONFERRONI)

 /CRITERIA = ALPHA(0.05)

 /DESIGN = TRT.

For one-way ANCOVA, with d 24–35 BWG as an example, where BWG (AVWGAIN2435) was defined as the dependent variable, treatments (TRT) as the independent variable, and d 24–35 male % (MD2435) as the covariate, the SPSS Syntax codes are:

UNIANOVA AVWGAIND2435 BY TRT WITH MD2435

 /METHOD = SSTYPE(3)

 /INTERCEPT = INCLUDE

 /EMMEANS = TABLES(OVERALL)WITH(MD2435 = MEAN)

 /EMMEANS = TABLES(TRT)WITH(MD2435 = MEAN) COMPARE ADJ(BONFERRONI)

 /CRITERIA = ALPHA(0.05)

 /DESIGN = MD2435 TRT.

The performance data from experiment 4 was analysed in a 2 × 3 factorial arrangement of treatments with male % as a covariate. Equality of error variances was performed on each set of data using Levene’s test at the level of *P* < 0.05. For the one-way and factorial ANOVA post hoc multiple comparisons for observed treatment means were performed using Tukey’s test if equal variances were assumed and Tamhanes T2 test if equal variances were not assumed. Treatment estimated marginal means were analysed as part of the one-way and factorial ANOVA and the treatment effects were compared using Bonferroni test for the factorial ANCOVA. Pearson correlation test was employed for correlation analysis between the dependent variable and covariate. Interaction between the covariate and independent variable was tested for each set of data at the level of *P* < 0.05. The statistics recorded for the ANOVA and ANCOVA were MSE, F-statistic, model significance, adjusted R^2^ and the observed power.

For a 2×3 factorial ANOVA with d 28–35 BWG as an example where BWG (WGbdd2835g) was defined as the dependent variable and the two treatment levels (Enzymes and sAXtAX) as the independent variables, the SPSS Syntax codes are:

UNIANOVA WGbdd2835g BY Enzymes sAXtAX

 /METHOD = SSTYPE(3)

 /INTERCEPT = INCLUDE

 /POSTHOC = Enzymes sAXtAX(TUKEY)

 /EMMEANS = TABLES(OVERALL)

 /EMMEANS = TABLES(Enzymes) COMPARE ADJ(BONFERRONI)

 /EMMEANS = TABLES(sAXtAX) COMPARE ADJ(BONFERRONI)

 /EMMEANS = TABLES(Enzymes*sAXtAX)

 /CRITERIA = ALPHA(0.05)

 /DESIGN = Enzymes sAXtAX Enzymes*sAXtAX.

For a 2×3 factorial ANCOVA with d 28–35 BWG as an example where BWG (WGbdd2835g) was defined as the dependent variable, the two treatment levels (Enzymes and sAXtAX) as the independent variables and d 28–35 male % (Sexcov. D35) as the covariate, the SPSS Syntax codes are:

UNIANOVA WGbdd2835g BY Enzymes sAXtAX WITH Sexcov.D35

 /METHOD = SSTYPE(3)

 /INTERCEPT = INCLUDE

 /EMMEANS = TABLES(OVERALL) WITH(Sexcov.D35 = MEAN)

 /EMMEANS = TABLES(Enzymes) WITH(Sexcov.D35 = MEAN) COMPARE ADJ(BONFERRONI)

 /EMMEANS = TABLES(sAXtAX) WITH(Sexcov.D35 = MEAN) COMPARE ADJ(BONFERRONI)

 /EMMEANS = TABLES(Enzymes*sAXtAX) WITH(Sexcov.D35 = MEAN)

 /CRITERIA = ALPHA(0.05)

 /DESIGN = Sexcov.D35 Enzymes sAXtAX Enzymes*sAXtAX.

## Results

### Experiment 1

The correlation data between the dependent variables and covariate for each phase in experiment 1 are presented in Table 1. There was no significant correlation (*P* > 0.05) between the male % and the dependent variables during d 0–10. The male % during d 10–24 had a significant negative correlation (*P* < 0.05) with FCR (r = -0.353) and significant positive correlations with BWG (*P* < 0.01, r = 0.414) and FI (*P* < 0.05, r = 0.357). During d 25–35, the male % had a significant positive correlation (*P* < 0.01) with BWG and FI (r = 0.455). There was no significant correlation (*P* > 0.05) between male % and FCR during d 25–35.

**Table 1:** Correlations between the independent variables and the covariate male % for each phase for Experiment 1.

Parameter	d 0–10	d 10–24	d 25–35
FCR	-0.06	-.353*	-0.09
BWG	0.01	.414**	.455**
FI	-0.01	.357*	.455**

**. Correlation is significant at the 0.01 level (2-tailed).

*. Correlation is significant at the 0.05 level (2-tailed).

The comparative statistics for a one-way ANOVA and ANCOVA during each phase in experiment 1 are presented in Tables 2 and 3. As there was no significant correlation between male % and the dependent variables during d 0–10, ANCOVA was not performed for this period. During d 10–24, ANCOVA reduced the MSE, increased the F-statistic, adjusted R^2^ value and observed power and improved the model significance for BWG, FI and FCR. The same results were achieved for BWG and FI during d 25–35; however, ANCOVA was not performed on FCR during this phase due to there being no significant correlation between the covariate and dependent variable.

**Table 2:** MSE, F-statistic and model significance of performance data when analysed by a one-way ANOVA and ANCOVA during different phases for Experiment 1.

		MSE			F-statistic			Model significance		
		ANOVA	ANCOVA	Change*	ANOVA	ANCOVA	Change*	ANOVA	ANCOVA	Change*
Day 10–24	BWG	2855	2168	-687	12.5	16.1	+3.60	1.89E-07	2.08E-09	-1.87E-07
	FI	3770	3355	-415	2.22	3.05	+0.89	0.08	0.02	-0.06
	FCR	6.90E-03	6.14E-03	-7.60E-04	7.92	8.46	+0.54	2.52E-05	5.37E-06	-1.98E-05
Day 25–35	BWG	4274	3284	-990	0.23	2.43	+2.20	0.95	0.04	-0.90
	FI	7407	5983	-1423	0.65	2.51	+1.85	0.66	0.04	-0.62

* indicating the value change by applying ANCOVA compared to ANOVA.

**Table 3:** Adjusted R^2^ and observed power of performance data when analysed by a one-way ANOVA and ANCOVA during different phases for Experiment 1.

		Adjusted R^2^			Observed power		
		ANOVA	ANCOVA	Change*	ANOVA	ANCOVA	Change*
Day 10–24	BWG	0.55	0.66	+0.11	1.00	1.00	+4E-06
	FI	0.11	0.21	+0.10	0.65	0.86	+0.21
	FCR	0.42	0.49	+0.06	0.999	1.00	+0.001
Day 25–35	BWG	-0.09	0.16	+0.25	0.10	0.76	+0.66
	FI	-0.04	0.16	+0.20	0.21	0.78	+0.56

* indicating the value change by applying ANCOVA compared to ANOVA.

### Experiment 2

The correlation data between the dependent variables and covariate for each phase in experiment 2 are presented in Table 4. There was no significant correlation (*P* > 0.05) between the covariate and dependent variables during d 0–10. From d 10–24, there was a significant positive correlation (*P* < 0.01) between BWG and male % (r = 0.396) but the covariate had no significant correlation with FI and FCR (*P* > 0.05) during this phase. From d 25–35, the male % had a significant positive correlation (*P* < 0.01) with FI (r = 0.508) and BWG (r = 0.602), but no significant correlation with FCR (*P* > 0.05).

**Table 4:** Correlations between the independent variables and the covariate male % for each phase for Experiment 2.

Parameter	d 0–10	d 10–24	d 25–35
FCR	-0.10	-0.27	-0.05
BWG	0.15	.396**	.508**
FI	0.11	0.26	.602**

**. Correlation is significant at the 0.01 level (2-tailed).

The comparative statistics for a one-way ANOVA and ANCOVA during each phase in experiment 2 are presented in Tables 5 and 6. As there was no significant correlation between male % and the dependent variables during d 0–10, ANCOVA was not performed for this period. ANCOVA was also not performed on the FCR data as there were no significant correlations between the covariate and this parameter for all the phases. From d 10–24, only the BWG data was analysed using male % as a covariate and this resulted in a decrease in MSE, an increase in the F-statistic, adjusted R^2^ value and observed power and an improvement in model significance. From day 25–35, the MSE of BWG and FI was reduced and the F-statistic was increased through the inclusion of male % as a covariate. There was also an improved model significance, model fit and observed power value for BWG and FI when ANCOVA was performed.

**Table 5:** MSE, F-statistic and model significance of performance data when analysed by a one-way ANOVA and ANCOVA during different phases for Experiment 2.

		MSE			F-statistic			Model significance		
		ANOVA	ANCOVA	Change*	ANOVA	ANCOVA	Change*	ANOVA	ANCOVA	Change*
Day 10–24	BWG	3568	2840	-727	0.80	2.79	+2.00	0.56	0.02	-0.53
Day 25–35	BWG	5135	3876	-1258	0.84	3.36	+2.53	0.53	0.01	-0.52
	FI	10034	6590	-3443	0.29	4.19	+3.90	0.92	0.002	-0.92

* indicating the value change by applying ANCOVA compared to ANOVA.

**Table 6:** Adjusted R^2^ and observed power of performance data when analysed by a one-way ANOVA and ANCOVA during different phases for Experiment 2.

		Adjusted R^2^			Observed power		
		ANOVA	ANCOVA	Change*	ANOVA	ANCOVA	Change*
Day 10–24	BWG	-0.02	0.19	+0.21	0.26	0.83	+0.57
Day 25–35	BWG	-0.02	0.23	+0.25	0.27	0.90	+0.63
	FI	-0.08	0.29	+0.37	0.11	0.96	+0.84

* indicating the value change by applying ANCOVA compared to ANOVA

### Experiment 3

The correlation data between the dependent variables and covariate for each phase in experiment 3 are presented in Table 7. There was no significant correlation between the covariate and dependent variables from d 0–10 (*P* > 0.05). From d 10–21, there was a significant positive correlation (*P* < 0.05, r = 0.287) between BWG and male % but the covariate had no significant correlation with FI and FCR (*P* > 0.05) during this phase. From d 21–35, the male % had a significant positive correlation with FI (*P* < 0.01, r = 0.430) and BWG (*P* < 0.01, r = 0.486) and a significant negative correlation with FCR (*P* < 0.05, r = -0.316).

**Table 7:** Correlations between the independent variables and the covariate male % for each phase for Experiment 3.

Parameter	d 0–10	d 10–21	d 21–35
FCR	-0.22	-0.20	-.316*
BWG	0.17	.287*	.430**
FI	0.01	0.24	.486**

**. Correlation is significant at the 0.01 level (2-tailed).

*. Correlation is significant at the 0.05 level (2-tailed).

**Fig 1:**
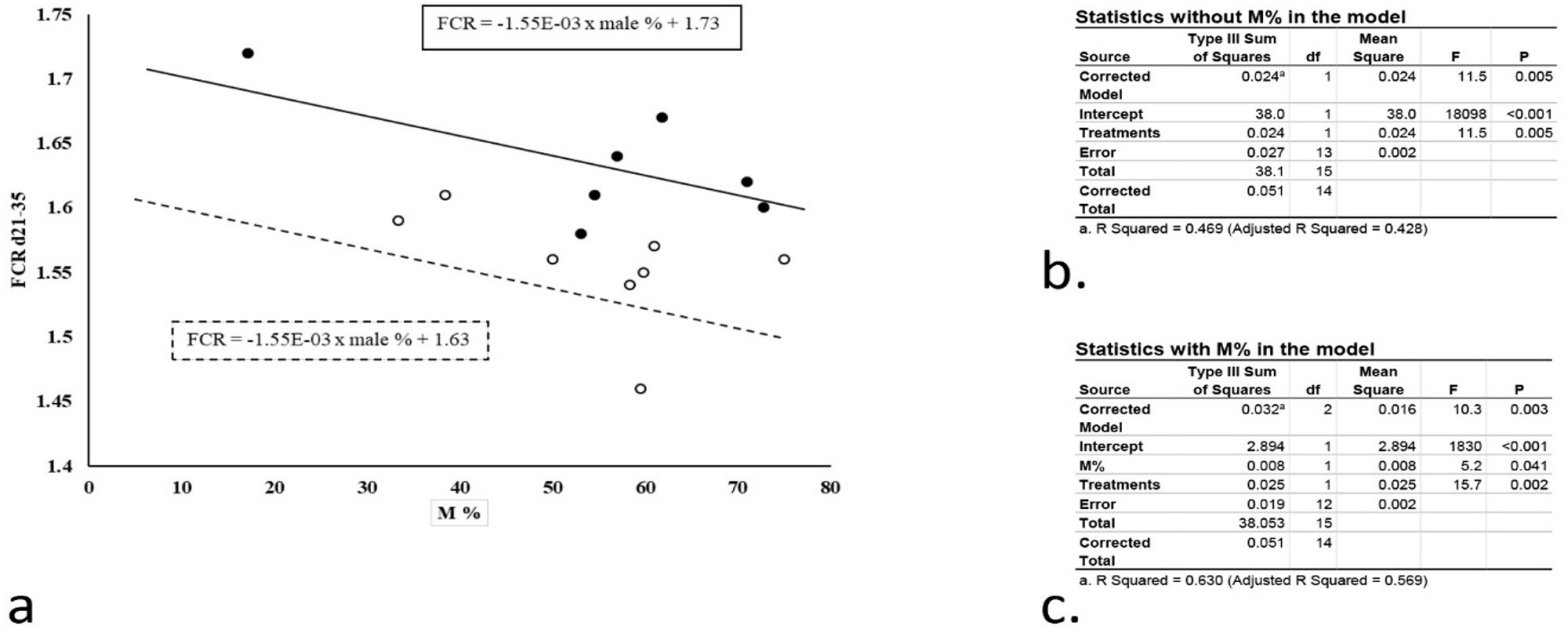
The benefit of the inclusion of male % as a covariate in the statistical analysis model. a. FCR during d 21–35 was plotted against the male %; b. the statistical analysis outcome without the inclusion of male % as a covariate; c. the statistical analysis outcome with the inclusion of male % as a covariate. "solid line" Treatment with antibiotic; "dashed line" Treatment with a mixture of additives; "filled circle" FCR of pens fed diet with antibiotic supplementation; "unfilled circle" FCR of pens fed diet with a mixture of additives supplementation.

The comparative statistics for a one-way ANOVA and ANCOVA during each phase in experiment 3 are presented in Tables 8 and 9. As there was no significant correlation between male % and the dependent variables during d 0–10, ANCOVA was not performed for this period. From d 10–21 only the BWG data was analysed using male % as a covariate, which resulted in a decrease in MSE, a decrease in the F-statistic, an increase in the adjusted R^2^ value and no change in observed power. The model significance was only slightly improved. From day 21–35, the MSE was reduced and the F-statistic was increased through the inclusion of male % as a covariate for all the parameters. There was also an improved model significance, model fit and observed power value for all the parameters. To demonstrate the beneficial effect of ANCOVA, we presented the results of two treatments from experiment 3 (T5 and T6), i.e., a group of birds fed diets supplemented with antibiotics and another supplemented with a mixture of additives from Experiment 3. The FCR during d 21–35 was plotted against the male % for each pen, (Fig 1a), and the statistics were analysed without (Fig 1b) and with (Fig 1c) male % in the model. It is clearly shown that male % was linearly correlated to the FCR for both treatments and the slopes of the equations are not different. Following the inclusion of male % in the statistical model, the P values of model fit decreased, and the F statistic and R^2^ increased suggesting the analysis of the data is more powerful. Furthermore, it was shown that the P value of the treatment effect was improved from 0.005 to 0.002 indicating the benefit of including the male % as a covariate in the model.

**Table 8:** MSE, F-statistic and model significance of performance data when analysed by a one-way ANOVA and ANCOVA during different phases for Experiment 3.

		MSE			F-statistic			Model significance		
		ANOVA	ANCOVA	Change*	ANOVA	ANCOVA	Change*	ANOVA	ANCOVA	Change*
Day 10–21	BWG	935	804	-131	54.2	53.8	-0.34	3.13E-17	7.02E-18	-2.43E-17
Day 21–35	BWG	6092	4750	-1342	0.73	2.93	+2.20	0.60	0.02	-0.59
	FI	10061	7475	-2587	1.78	4.59	+2.81	0.14	0.001	-0.14
	FCR	1.40E-03	1.20E-03	-2.00E-04	4.41	5.80	+1.39	2.72E-03	2.17E-04	-2.50E-03

* indicating the value change by applying ANCOVA compared to ANOVA.

**Table 9:** Adjusted R^2^ and observed power of performance data when analysed by a one-way ANOVA and ANCOVA during different phases for Experiment 3.

		Adjusted R^2^			Observed power		
		ANOVA	ANCOVA	Change*	ANOVA	ANCOVA	Change*
Day 10–21	BWG	0.85	0.87	+0.02	1.00	1.00	+0.00
Day 21–35	BWG	-0.03	0.20	+0.23	0.24	0.85	+0.61
	FI	0.08	0.31	+0.24	0.56	0.97	+0.42
	FCR	0.28	0.39	+0.12	0.94	0.99	+0.05

* indicating the value change by applying ANCOVA compared to ANOVA.

### Experiment 4

The correlation data between the dependent variables and covariate for each phase in experiment 4 are presented in Table 10. The male % during d 19–28 had a significant negative correlation (*P* < 0.01) with BWG (r = -0.447) and FI (r = -0.370). During d 28–35 and d 19–35, the male % had a significant positive correlation (*P* < 0.01) with BWG and FI. There was no significant correlation (*P* > 0.05) between male % and FCR for all phases.

**Table 10:** Correlations between the independent variables and the covariate male % for each phase for Experiment 4.

Parameter	d 19–28	d 28–35	d 19–35
FCR	0.04	-0.10	-0.10
BWG	-.447**	.479**	.560**
FI	-.370**	.388**	.482**

**. Correlation is significant at the 0.01 level (2-tailed).

*. Correlation is significant at the 0.05 level (2-tailed).

The comparative statistics for a 2 × 3 factorial ANOVA and ANCOVA during each phase in experiment 4 are presented in Tables 11 and 12. During all 3 phases the MSE of BWG and FI were reduced and the F-statistics increased by including male % as a covariate. There was also an improved model significance, model fit and observed power value for BWG and FI when ANCOVA was performed. As there were no significant correlations between male % and FCR for this experiment, ANCOVA was not applied to this parameter.

**Table 11:** MSE, F-statistic and model significance of performance data when analysed by a one-way ANOVA and ANCOVA during different phases for Experiment 4.

		MSE			F-statistic			Model significance		
		ANOVA	ANCOVA	Change*	ANOVA	ANCOVA	Change*	ANOVA	ANCOVA	Change*
Day 19–28	BWG	19.6	14.4	-5.20	3.25	6.39	+3.14	0.01	8.31E-05	-1.39E-02
	FI	56.5	49.4	-7.07	1.71	2.80	+1.09	0.15	0.02	-1.31E-01
Day 28–35	BWG	53.7	42.3	-11.3	0.15	2.20	+2.05	0.98	0.06	-9.17E-01
	FI	128	111	-15.9	0.70	1.83	+1.13	0.63	0.12	-5.09E-01
Day 19–35	BWG	23.3	15.5	-7.76	0.89	4.80	+3.90	0.49	8.75E-04	-4.93E-01
	FI	49.9	39.0	-10.9	1.37	3.60	+2.22	0.25	5.91E-03	-2.48E-01

* indicating the value change by applying ANCOVA compared to ANOVA.

**Table 12:** Adjusted R^2^ and observed power of performance data when analysed by a one-way ANOVA and ANCOVA during different phases for Experiment 4.

		Adjusted R^2^			Observed power		
		ANOVA	ANCOVA	Change*	ANOVA	ANCOVA	Change*
Day 19–28	BWG	0.19	0.41	+0.21	0.85	0.10	+0.15
	FI	0.07	0.19	+0.12	0.53	0.83	+0.29
Day 28–35	BWG	-0.10	0.13	+0.23	0.08	0.71	+0.63
	FI	-0.03	0.10	+0.13	0.23	0.62	+0.39
Day 19–35	BWG	-0.01	0.33	+0.34	0.29	0.98	+0.69
	FI	0.04	0.25	+0.21	0.44	0.92	+0.48

* indicating the value change by applying ANCOVA compared to ANOVA.

## Discussion

In the current study, the inclusion of sex proportion (i.e., male %) as a covariate in the statistical analysis during each phase of 4 separate experiments using mixed-sex broilers with unequal numbers of male and female birds in each pen was evaluated and compared against ANOVA analysis. The outcome of this study indicated that the use of sex proportion as a covariate in ANCOVA significantly improved the statistical test power for performance measurements in broilers which are unevenly distributed according to sex within each experimental unit, particularly during the later stages of growth. These results support the hypothesis that ANCOVA using male % as a covariate will result in more robust data from experiments that make use of mixed-sex broilers without the need for more animals.

The data from all 4 experiments met the assumptions for running an ANCOVA at least during the later stages of growth. During some phases, there was no significant correlation between the dependent variable and the covariate, which occurred during the starter phases (d 0–10) for experiments 1–3. Due to this assumption violation, ANCOVA was not applied for this period for these three experiments. Marks [14] reported that sexual dimorphism is less pronounced during the first week of growth in broilers and this may have led to the lack of a correlation between male % and performance parameters during d 0–10. For experiments 1 and 2, the birds were feather sexed prior to placement and were therefore allocated to pens with the same proportion of males and females. This constant sex proportion across the pens also led to the lack of correlation during the starter phase.

The results support the argument that all the assumptions need to be met first in order for ANCOVA to be used to account for the errors introduced due to the sex effect so as to increase the power of analysis. In this study, the lack of a significant correlation between the covariate and the dependent variables in the early feeding phases meant that in most cases, the inclusion of sex proportion as a covariate does not result in any improvement in the measured statistics parameters; thus, ANCOVA is not needed for such data. In addition to this, the fact that there was already a high model significance and observed power when ANOVA was performed, such as was the case in the grower period for experiments 1 and 3, where the treatment effects were highly significant, then the inclusion of male % as a covariate will result in very little improvements in model significance and increases in observed power. So it can be concluded that if a high level of model significance and observed power are already observed, for example, *P* < 0.001, then ANCOVA will not be necessary as whether ANCOVA or ANOVA is performed, the same result will be achieved. It is also important to note that in all the experiments, the FCR response to ANCOVA was not in line with the response of BWG and FI. This is because the FCR response to the sex of the broilers is a ratio between FI and BWG, thus a linear model may not apply to the effect of sex on FCR. Therefore, a different analysis model may be needed in order for any potential improvements to be achieved.

Interestingly, the inclusion of the sex effect in the statistical model when both sexes are used has been very scarce, particularly in poultry nutrition studies. One of the reasons may be that researchers tend to include sex, i.e., male and female, as a variable. However, it cannot be included as a covariate as it is a categorical variable. A way in which sex can be used as a categorical variable, and to make sure the variation introduced into the model is accounted for, is by including sex as a blocking variable [15]. However, the use of sex as a blocking variable will reduce the sample size and will only help when the parameters are measured separately with male and female animals. In the case of randomly distributed as-hatched broilers without sexing, the block effect cannot be used in the model and sex would need to be transformed into a continuous variable for use as a covariate. We used the male % of each pen, a continuous variable, as a covariate thereby allowing it to be included in the ANCOVA model whereby it effectively helped improve the statistical power of the analysis.

On the other hand, if the study aims to determine the effect sex has on dependent variables, sex as a categorical variable should be used as the main effect, and the allocation of animals in the treatments would have to be according to their sex. Some authors have not made it clear in their studies about whether they have used sex as a covariate or as a main effect. It is important to differentiate between these two variables, as they are very different and yet have been used interchangeably in animal and human studies. Duffy and Epperson [16], evaluated 251 neuropsychopharmacology studies done in humans that included both males and females, where sex was thought to have an impact on the measurements. However, a total of 80% of these studies included the proportion of sex as a covariate which could not determine the different responses between males and females but rather eliminated the variation due to the sex effect. It was demonstrated that only the other 20% included sex as a main effect to correctly test for sex differences. A few published studies with mixed-sex chickens used sex as a covariate in the statistical analysis [17–19]. However, the experimental designs of Payne and Southern [18] and Van der Klein, et al. [19] did not warrant the benefit of using sex as a covariate. Payne and Southern [18] sexed the broilers on d 0 to investigate the effect of selenium from different sources. The birds were allocated to each treatment with 4 replicates of 50 male broilers per replicate pen and 3 replicates of 55 female broilers per replicate. Such a design suggests sex should have been used as a main effect or blocking variable rather than a covariate. Van der Klein, et al. [19] used mixed-sex broilers to determine the effect of quantitative feed restriction on allometric growth. The birds were sexed on d 0 and 5 birds each of male and female were allocated to each pen of 8 different treatments with 4 replicates each. For the analysis of the performance data (FI, BW and FCR), the proportion of males in each pen as a covariate was included in the model. Despite the fact that the proportion of males was a proper covariate, the use of covariate analysis may not be justified due to the equal distribution of male and female chickens in each pen. There was also presumably no correlation between the measured variables and the proportion of males which violates the assumption of there having to be a significant correlation between variables and covariates for the ANCOVA model. Johnsson et al. [17] used female and male chickens of a segregation population for quantitative trait locus mapping study, and sex was used as a covariate to accommodate for the sex effect on the traits of individual chickens. This study recorded the measurements of individual chickens rather than pen-based measurements, therefore this experiment is not relevant to the topic under discussion in the current study.

The majority of poultry nutritional studies have ignored possible variations introduced by the unequal distribution of birds according to sex in experiments by analysis the data using ANOVA rather than ANCOVA [3, 20]. This presumably resulted in less powerful outcomes, and in some cases, misleading conclusions may be drawn. This makes it important to highlight that ANCOVA using sex proportion as a covariate should be performed to reduce bias and improve observed power when the sex effect is significant for the parameters of interest in research. On the other hand, it should also be noted that the use of sex proportion as a covariate does not necessarily always reduce the P values or increase the treatment significance per se however it is certain that the test power and model fit are always enhanced. In the cases where the null hypothesis stands, i.e., the treatment does not lead to a significant difference, the covariate inclusion would increase the P-value and thus make the difference less significant.

Currently, the majority of farms across in Australia are rearing male and female broilers together for chicken meat production [21]. However, the nutritional and possibly health research data obtained in research studies has been mainly based on male broilers due to the fact that the experiments using single-sex birds show smaller variations compared to using mixed-sex birds which are unevenly distributed according to sex across pens. This may lead to a bias in the results which are used in the industry, for example, to determine the optimal nutrient requirements for broilers reared under farming settings. The use of ANCOVA in the statistical analysis for nutritional studies, with sex proportion as a covariate will provide an opportunity for the researchers to use mixed-sex birds without having to sex birds on the day of arrival. This is particularly relevant under the current situation that feather sexing is no longer possible, and other approaches for sexing day-old chickens are not available or are costly. This approach helps researchers to obtain more industry-relevant data without the need to increase the scale of the study in order to achieve adequate test powers by using more replicates and thus more animals.

To the best of our knowledge, the current study is the first paper that takes into consideration the variations introduced by using mixed-sex broilers that are unevenly distributed within the experimental units, i.e., pens or cages. We have used ANCOVA to performance data with male % as a covariate in the statistical analysis. Our results show that ANCOVA can help to improve the experimental power and reduce the MSE in performance trials provided all the assumptions for ANCOVA are met, and treatment effects are not already highly significant. With researchers finding it increasingly difficult to source sexed day-old chicks for use in experiments, mixed-sex birds have to be used in the experiments for nutritional and health-related studies. We anticipate the outcomes of the current study will be applied in future experiments that make use of mixed-sex broilers so as to ensure a future generation of meaningful and robust data with no need to increase the number of animals used.

## Supporting information

S1 Table Comparison of tests of between-subjects effects for body weight gain during d 25–35 when data was analysed by ANOVA and ANCOVA in Experiment 1. (DOCX)

S2 Table Comparison of grand means and standard error for body weight gain during d 25–35 when data was analysed by ANOVA and ANCOVA in Experiment 1. (DOCX)

S3 Table Comparison of tests of between-subjects effects for body weight gain during d 25–35 when data was analysed by ANOVA and ANCOVA in Experiment 2. (DOCX)

S4 Table Comparison of grand means and standard error for body weight gain during d 25–35 when data was analysed by ANOVA and ANCOVA in Experiment 2. (DOCX)

S5 Table Comparison of tests of between-subjects effects for body weight gain during d 21–35 when data was analysed by ANOVA and ANCOVA in Experiment 3. (DOCX)

S6 Table Comparison of grand means and standard error for body weight gain during d 21–35 when data was analysed by ANOVA and ANCOVA in Experiment 3. (DOCX)

S7 Table Comparison of tests of between-subjects effects for body weight gain during d 28–35 when data was analysed by ANOVA and ANCOVA in Experiment 4. (DOCX)

S8 Table Comparison of grand means and standard error for body weight gain during d 28–35 when data was analysed by ANOVA and ANCOVA in Experiment 4. (DOCX)
