# Supplementary material for: Sex proportion as a covariate increases the statistical test power in growth performance based experiments using as-hatched broilers
Source: PLoS One. 2023 Jan 20;18(1):e0280040. doi: 10.1371/journal.pone.0280040 (PMC9857968; doi:10.1371/journal.pone.0280040)
Supplement: S1 Table — (DOCX) [file pone.0280040.s001.docx]

**Appendix Table 1** Comparison of tests of between-subjects effects for body weight gain during d 25-35 when data was analysed by ANOVA and ANCOVA in Experiment 1

| Source | Type III Sum of Squares | | df | | Mean square | | F-value | | Significance | |
| --- | --- | --- | --- | --- | --- | --- | --- | --- | --- | --- |
|  | ANOVA | ANCOVA | ANOVA | ANCOVA | ANOVA | ANCOVA | ANOVA | ANCOVA | ANOVA | ANCOVA |
| Corrected Model | 5003 | 47895 | 5 | 6 | 1001 | 7982 | 0.23 | 2.43 | 0.95 | 0.04 |
| Intercept | 61533555 | 7115107 | 1 | 1 | 61533555 | 7115107 | 14395 | 2166 | 8.9445E-53 | 8.62E-36 |
| M % | . | 42892 | . | 1 | . | 42892 | . | 13.1 | . | 8.52E-04 |
| Treatments | 5003 | 11455 | 5 | 5 | 1001 | 2291 | 0.23 | 0.70 | 0.95 | 0.63 |
| Error | 170984. | 128093 | 40 | 39 | 4275 | 3284 |  |  |  |  |
| Total | 62422526 | 62422526 | 46 | 46 |  |  |  |  |  |  |
| Corrected Total | 175987 | 175987 | 45 | 45 |  |  |  |  |  |  |
