# Supplementary material for: Sex proportion as a covariate increases the statistical test power in growth performance based experiments using as-hatched broilers
Source: PLoS One. 2023 Jan 20;18(1):e0280040. doi: 10.1371/journal.pone.0280040 (PMC9857968; doi:10.1371/journal.pone.0280040)
Supplement: S3 Table — (DOCX) [file pone.0280040.s003.docx]

**Appendix Table 3** Comparison of tests of between-subjects effects for body weight gain during d 25-35 when data was analysed by ANOVA and ANCOVA in Experiment 2

| Source | Type III Sum of Squares | | df | | Mean square | | F-value | | Significance | |
| --- | --- | --- | --- | --- | --- | --- | --- | --- | --- | --- |
|  | ANOVA | ANCOVA | ANOVA | ANCOVA | ANOVA | ANCOVA | ANOVA | ANCOVA | ANOVA | ANCOVA |
| Corrected Model | 21511 | 78251 | 5 | 6 | 4302 | 13042 | 0.84 | 3.36 | 0.53 | 8.66E-03 |
| Intercept | 56963562 | 2734650 | 1 | 1 | 56963562 | 2734650 | 11092 | 705 | 1.57E-52 | 1.866E-27 |
| M % | . | 56740 | . | 1 | . | 56740 | . | 14.6 | . | 4.37E-04 |
| Treatments | 21511 | 17024 | 5 | 5 | 4302 | 3405 | 0.84 | 0.88 | 0.53 | 0.50 |
| Error | 215689 | 158950 | 42 | 41 | 5136 | 3877 |  |  |  |  |
| Total | 57444171 | 57444171 | 48 | 48 |  |  |  |  |  |  |
| Corrected Total | 237201 | 237201 | 47 | 47 |  |  |  |  |  |  |
