# Supplementary material for: Sex proportion as a covariate increases the statistical test power in growth performance based experiments using as-hatched broilers
Source: PLoS One. 2023 Jan 20;18(1):e0280040. doi: 10.1371/journal.pone.0280040 (PMC9857968; doi:10.1371/journal.pone.0280040)
Supplement: S4 Table — (DOCX) [file pone.0280040.s004.docx]

**Appendix Table 4** Comparison of grand means and standard error for body weight gain during d 25-35 when data was analysed by ANOVA and ANCOVA in Experiment 2

| Mean | | Standard error | |  |  |  |  |  |
| --- | --- | --- | --- | --- | --- | --- | --- | --- |
| ANOVA | ANCOVA | ANOVA | ANCOVA |  |  |  |  |  |
| 1092.30 | 1092.31* | 10.4 | 9.01 |  |  |  |  |  |
| * Covariates appearing in the model are evaluated at the following values: M% = 43.810 | | | | | | | | |
