# Supplementary material for: Sex proportion as a covariate increases the statistical test power in growth performance based experiments using as-hatched broilers
Source: PLoS One. 2023 Jan 20;18(1):e0280040. doi: 10.1371/journal.pone.0280040 (PMC9857968; doi:10.1371/journal.pone.0280040)
Supplement: S5 Table — (DOCX) [file pone.0280040.s005.docx]

**Appendix Table 5** Comparison of tests of between-subjects effects for body weight gain during d 21-35 when data was analysed by ANOVA and ANCOVA in Experiment 3

| Source | Type III Sum of Squares | | df | | Mean square | | F-value | | Significance | |
| --- | --- | --- | --- | --- | --- | --- | --- | --- | --- | --- |
|  | ANOVA | ANCOVA | ANOVA | ANCOVA | ANOVA | ANCOVA | ANOVA | ANCOVA | ANOVA | ANCOVA |
| Corrected Model | 22290 | 83411 | 5 | 6 | 4458 | 13902 | 0.73 | 2.93 | 0.60 | 0.02 |
| Intercept | 102931212 | 7936484 | 1 | 1 | 1.03E+08 | 7936484 | 16896 | 1671 | 2.35E-56 | 7.476E-35 |
| M % | . | 61121 | . | 1 | . | 61121 | . | 12.9 | . | 8.83E-04 |
| Treatments | 22290 | 32074 | 5 | 5 | 4458 | 6415 | 0.73 | 1.35 | 0.60 | 0.26 |
| Error | 255864 | 194743 | 42 | 41 | 6092 | 4750 |  |  |  |  |
| Total | 103209366 | 103209366 | 48 | 48 |  |  |  |  |  |  |
| Corrected Total | 278154 | 278154 | 47 | 47 |  |  |  |  |  |  |
