# Supplementary material for: Sex proportion as a covariate increases the statistical test power in growth performance based experiments using as-hatched broilers
Source: PLoS One. 2023 Jan 20;18(1):e0280040. doi: 10.1371/journal.pone.0280040 (PMC9857968; doi:10.1371/journal.pone.0280040)
Supplement: S7 Table — (DOCX) [file pone.0280040.s007.docx]

**Appendix Table 7** Comparison of tests of between-subjects effects for body weight gain during d 28-35 when data was analysed by ANOVA and ANCOVA in Experiment 4

| Source | Type III Sum of Squares | | df | | Mean square | | F-value | | Significance | |
| --- | --- | --- | --- | --- | --- | --- | --- | --- | --- | --- |
|  | ANOVA | ANCOVA | ANOVA | ANCOVA | ANOVA | ANCOVA | ANOVA | ANCOVA | ANOVA | ANCOVA |
| Corrected Model | 40.1 | 559 | 5 | 6 | 8.03 | 93.2 | 0.15 | 2.20 | 0.98 | 0.06 |
| Intercept | 526408 | 37077 | 1 | 1 | 526408 | 37077 | 9808 | 876 | 2.06E-51 | 2.73E-29 |
| M% | . | 519 | . | 1 | . | 519 | . | 12.3 | . | 1.13E-03 |
| Enzymes | 21.7 | 12.5 | 1 | 1 | 21.7 | 12.5 | 0.40 | 0.30 | 0.53 | 0.59 |
| sAXtAX | 7.86 | 17.9 | 2 | 2 | 3.93 | 8.96 | 0.07 | 0.21 | 0.93 | 0.81 |
| Enzymes × sAXtAX | 10.6 | 1.95 | 2 | 2 | 5.31 | 0.98 | 0.10 | 0.02 | 0.91 | 0.98 |
| Error | 2254 | 1735 | 42 | 41 | 53.7 | 42.3 |  |  |  |  |
| Total | 528702 | 528702 | 48 | 48 |  |  |  |  |  |  |
| Corrected Total | 2294 | 2294 | 47 | 47 |  |  |  |  |  |  |
